# Supplementary material for: Resolving the full spectrum of human genome variation using Linked-Reads
Source: Genome Res. 2019 Apr;29(4):635–45. doi: 10.1101/gr.234443.118 (PMC6442396; doi:10.1101/gr.234443.118)
Supplement: Supplemental Material [file supp_gr.234443.118_Supplemental_longranger-2.2.zip › longranger-2.2.tar.bz2!/lr2.2-public/lib/stan/cmdstan/stan/doc/api/html/functions_2promote__scalar__type_8hpp_source.html]

Stan: src/stan/math/functions/promote\_scalar\_type.hpp Source File


|  |  |
| --- | --- |
|  | Stan  2.5.0  probability, sampling & optimization |


- Main Page
- Related Pages
- Namespaces
- Classes
- Files

- File List
- File Members

All Classes Namespaces Files Functions Variables Typedefs Enumerations Enumerator Friends Macros Pages

- src
- stan
- math
- functions

promote\_scalar\_type.hpp

Go to the documentation of this file.

1 #ifndef STAN\_\_MATH\_\_FUNCTIONS\_\_PROMOTE\_SCALAR\_TYPE\_HPP

2 #define STAN\_\_MATH\_\_FUNCTIONS\_\_PROMOTE\_SCALAR\_TYPE\_HPP

3

4 #include <vector>

5

6 namespace stan {

7

8  namespace math {

9

10

18  template <typename T, typename S>

19  struct promote\_scalar\_type {

20

24  typedef T type;

25

26  };

27

28

37  template <typename T, typename S>

38  struct promote\_scalar\_type<T, std::vector<S> > {

39

43  typedef std::vector<typename promote\_scalar\_type<T,S>::type> type;

44

45  };

46

47

48  }

49

50 }

51

52 #endif

stan::math::promote\_scalar\_type

Template metaprogram to calculate a type for converting a convertible type.

**Definition:** promote\_scalar\_type.hpp:19

stan::math::promote\_scalar\_type::type

T type

The promoted type.

**Definition:** promote\_scalar\_type.hpp:24

stan::math::promote\_scalar\_type< T, std::vector< S > >::type

std::vector< typename promote\_scalar\_type< T, S >::type > type

The promoted type.

**Definition:** promote\_scalar\_type.hpp:43


---

[ Stan Home Page ]

*© 2011–2014,
Stan Development Team.*
